# Microanalysis of Worn Surfaces of Selected Rotating Parts of an Internal Combustion Engine

**DOI:** 10.3390/ma15010158

**Published:** 2021-12-26

**Authors:** Rastislav Bernát, Jozef Žarnovský, Ivan Kováč, Rastislav Mikuš, Jiří Fries, Radoslav Csintalan

**Affiliations:** 1 Faculty of Engineering, Slovak University of Agriculture in Nitra, Tr. A. Hlinku 2, 949 76 Nitra, Slovakia; rastislav.bernat@uniag.sk (R.B.); ivan.kovac@uniag.sk (I.K.); rastislav.mikus@uniag.sk (R.M.); xcsintalan@uniag.sk (R.C.); 2 Faculty of Mechanical Engineering, Technical University of Ostrava, 17. listopadu 2172/15, 708 00 Ostrava-Poruba, Czech Republic; jiri.fries@vsb.cz

**Keywords:** cam, camshaft, crankshaft, roughness, microhardness

## Abstract

The present paper analyzes the damage of surfaces at spots of frictional contact, namely, the friction nodes on a camshaft and the connecting rod pins of a crankshaft. The resulting wear of the monitored friction nodes reduces the technical life of the machines, which can lead to the decommissioning of the machine. Wear was assessed by measuring roughness and microhardness and by observing the microstructures of the materials. The results of the experiments show that the rotating parts displayed visible wear on the cams, as well as on the connecting rod pins. The experiments revealed that wear was caused by the heating of the material to a high temperature during the operation of the machine and that there was a gradual cooling and tempering of the material, which led to a reduction in the microhardness of the monitored object. Lower microhardness values can be a cause of greater wear of the monitored objects. When comparing the microhardness of the used and the new camshaft, the hardened layer of the new camshaft from secondary production has a significantly smaller thickness compared to worn cams, which leads to the finding of a different material quality compared to the original parts from primary production. This fact indicates that the wear of a new camshaft as a spare part can contribute to the shortening of the technical life of friction nodes.

## 1. Introduction

Today, vehicles whose parts are powered by an internal combustion engine, which is exposed to various types of wear during the life cycle, are part of everyday life. Due to the rapidly increasing pace of industrial development in various sectors, greater demands are also placed on automotive parts. As the operating parameters increase, the undesirable effects of wear often worsen [1]. Increasing the performance of vehicles increases the problems of friction and wear to such an extent that, in some cases, we find ourselves on the verge of applicability in terms of material properties [2]. When two bodies interact, repeated local loading can lead to contact fatigue of the material; this is manifested by the peeling and chipping of particles from the surface, which is known as pitting [3].

**Figure 1:**
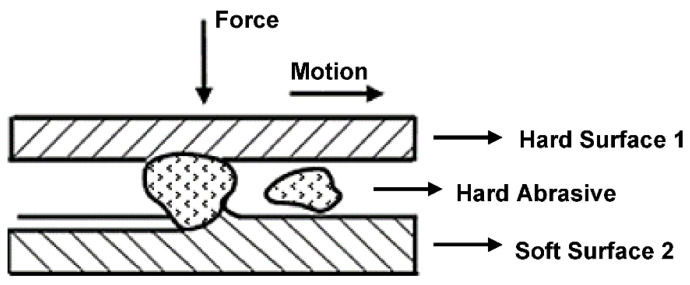
The principle of abrasive wear.

The cause of fatigue wear is the propagation of subsurface cracks caused by cyclic stress of the surface [4,5]. These cracks further branch and extend along the grain boundaries, where cavities are formed parallel to the surface, peeling of the material occurs and typical pits form. Contact fatigue is affected by various factors, such as speed, load, material, temperature, surface geometry, the lubricant used and the rate of friction [6,7,8]. For example, Dobrenizki et al. [9] states in his work that abrasive wear is more pronounced when using mineral motor oil. Pitting is demonstrated in the lubricating medium by the presence of characteristic flake particles of the damaged surface. The principle of abrasive wear is shown in Figure 1 [10].

The most common reasons for excessive wear of the monitored friction nodes are a lower lubrication quality, a too low oil viscosity and a too high or too low oil temperature, as well as a pressure drop in the lubrication system [11]. The next most possible causes of observed wear are the correct choice of materials at friction points, the choice of lubricant and its regular replacement and the operating conditions under which the engine runs (e.g., urban operation with frequent starting and driving with a cold engine or driving on the motorway) [12,13,14]. Valve timing is one of the key systems of an internal combustion engine, which contributes significantly to friction losses, especially under boundary and mixed lubrication conditions at lower crankshaft speeds.

Wear can be investigated by various experimental methods, which are divided according to the type of wear examined. To measure the magnitude of wear, various methods of surface analysis and quantities are used. There are direct methods that determine the absolute values of wear. The direct method measures quantities such as changes in the geometry of the sample, changes in the weight and the magnitude of the wear and tear of the material [15].

In today’s age of modern technologies, methods of contactless measurement are increasingly used. One of these methods is 3D scanning of the surface of the analyzed part [16]. The device used captures surface details of less than 0.2 mm. Surface irregularities can be automatically smoothed out by 3D scanning, and a 3D model of the analyzed surface can be created, from which it is possible to determine the places with the greatest wear. The monitored components are stressed by various variable forces, which further affect the wear in the friction nodes.

The goal of our study is to analyze the wear of the camshaft and crankshaft in terms of material change at the friction joints. Our analysis represents a comprehensive solution for monitoring a functional pair with respect to the aspects of roughness, microhardness and microstructure, and can also serve as an experimental method in determining the wear of the friction node of the functional pairs of an internal combustion engine. The technical life of the friction pairs of an internal combustion engine is influenced by many factors that can affect the wear of the friction nodes. Therefore, we focus our experiments on monitoring wear in terms of roughness and microhardness and the observation of the microstructures of materials at the point of wear of friction nodes.

The paper presents the results of monitoring the wear of the camshaft and connecting rod pins of the crankshaft from the point of view of roughness and microhardness of the material.

In the first phase of the experiments, we chose the correct spot of sampling collection, which was then analyzed in terms of roughness and microhardness, where the samples were prepared for experiments using a standard method for metallographic analysis. In the second phase, we subjected the prepared samples to microhardness analysis. When designing experiments to monitor the microhardness of individual shaft cams, we supplemented the measurements with microhardness measurements of a new shaft from secondary production, where the same material composition and shaft surface treatment were assumed. This was not confirmed by experiments where the new secondary camshaft material showed lower hardness. We also analyzed measurements of microhardness from the point of view of mathematical expression using regression dependences for individual cylinders, where the analysis of regressions showed a different microhardness of the used and new cams, which is evidenced by different material. Based on all the results from the experiments, it is possible to expect more complex information, especially in terms of roughness and microhardness of the materials used for functional pairs of friction nodes.

## 2. Materials and Methods

The monitored rotating parts were part of the engine Skoda 1.4 MPi, which is an atmospheric unit with two valves per cylinder. An increase in volume to 1397 cm^3^ was achieved by increasing the stroke to 78 mm while maintaining a 75.5 mm bore. The torque value for a given motor type is 120 Nm at 2500 m^−1^. The engine was in normal operation and regularly serviced at the intervals specified by the manufacturer. The experiments were performed after driving 325,142 km in operation. Table 1 shows the parameters of the monitored internal combustion engine.

### 2.1. Characteristics of Monitored Engine Components

#### 2.1.1. Camshaft Material

Steel C55 is used for processing with the chemical composition given in Table 2. The mechanical properties of the material are given in Table 3. Structural steel is suitable for turbocharger shafts, carousels, bent and other shafts, gears and rings, press pistons, piston rods, spindles, pins, lamellas, couplings, fuses, latches, holders, screws, levers, abnormal chain plates, various connecting parts etc. The weldability of steel is difficult.

#### 2.1.2. Crankshaft Material

Steel 37Cr4 is well malleable when hot and well machined in the annealed state with good hardenability up to a diameter of 40 mm. It is used for medium-stressed aircraft engine components and motor vehicle components. The chemical composition and mechanical properties of the material are given in Table 4 and Table 5.

### 2.2. Characteristics of Meters and Equipment

#### 2.2.1. Roughness Measurement

**Figure 2:**
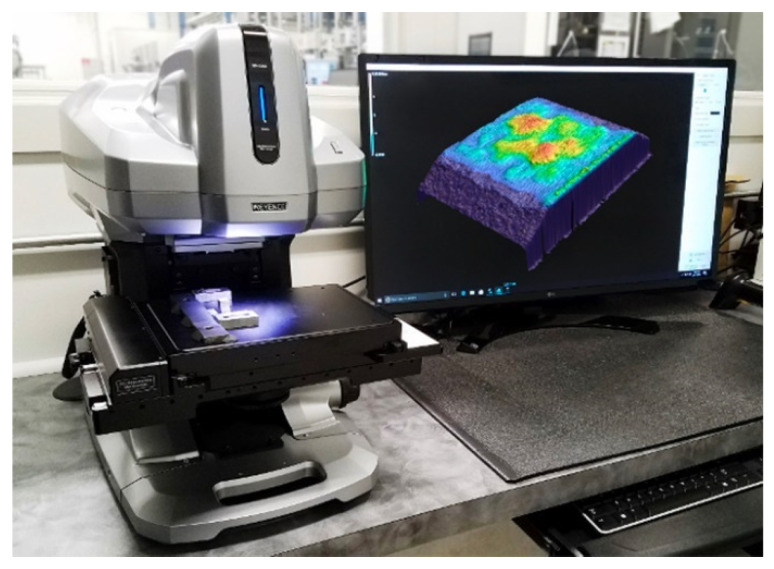
Confocal microscope with laser 3D scanning type Keyence VK-X 1050.

To monitor the roughness of the analyzed friction surfaces, we chose a confocal non-contact microscope with laser 3D scanning of the Keyence VK-X 1050 (Keyence, Osaka, Japan) type shown in Figure 2. The surface roughness represents the height of the unevenness of the real surface with respect to the perfect and ideally smooth surface. The resulting inequalities may be due to the production technology used or other influences, e.g., wear. The setting parameters used were standard for the monitored material according to the standard ISO 25178.

#### 2.2.2. Microhardness Measurement

**Figure 3:**
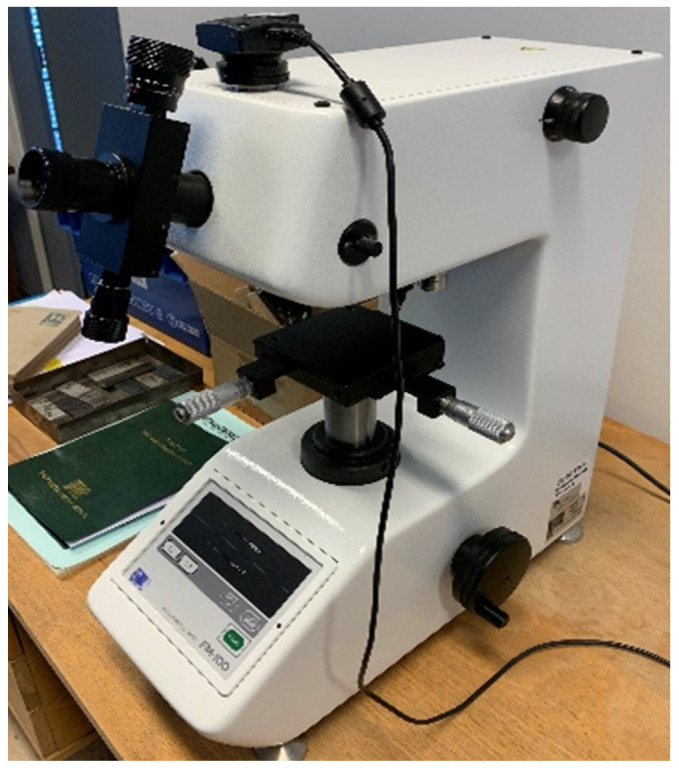
Microhardness tester Future-Tech FM 100.

The Future-Tech FM 100 (FUTURE-TECH CORP., Kawasaki, Japan) microhardness tester was used for microhardness measurements (Figure 3). The device uses the Vickers microhardness measurement method. The FM 100 microhardness tester has manual focusing, manual imprint measurement and automatic imprint creation according to a set value and a load exposure time from 5 to 40 s.

### 2.3. Measurement Characteristics

The correct determination of the sampling spot is important for the objective evaluation of the microstructure of the monitored material. The sampling spot is chosen according to the nature of the experiments, according to the specific requirements for evaluating the structure of the material, according to the method of production and according to the shape of the part and the external dimensions.

Sampling must be carried out in such a way that the condition is observed, namely, that the structure in the monitored metallographic section must not be thermally or mechanically affected. The samples taken must be characteristic of the material, both in terms of chemical composition and in terms of physical properties, which differ according to the production technology.

**Figure 4:**
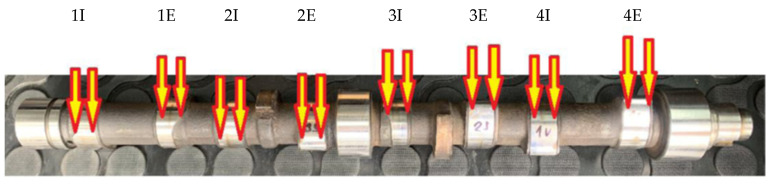
Camshaft sampling spots (I—intake cam, E—exhaust cam).

In our case, these are components heat treated by hardening, where the structure on the surface and in the core of the material is evaluated. Based on the mentioned experience, we chose the place of sampling from the camshaft on the nose of the cam, where the maximum visible wear appeared clearly (Figure 4).

In the experiments, we performed 5 measurements and evaluated the arithmetic mean. The variance of these values was up to 5%. We made the sampling on the nose of the cam where the maximum visible wear clearly appeared. For the objectivity of the measurement (thermal influence at the cutting point), it was not possible to carry out more provable measurements at the given place.

**Figure 5:**
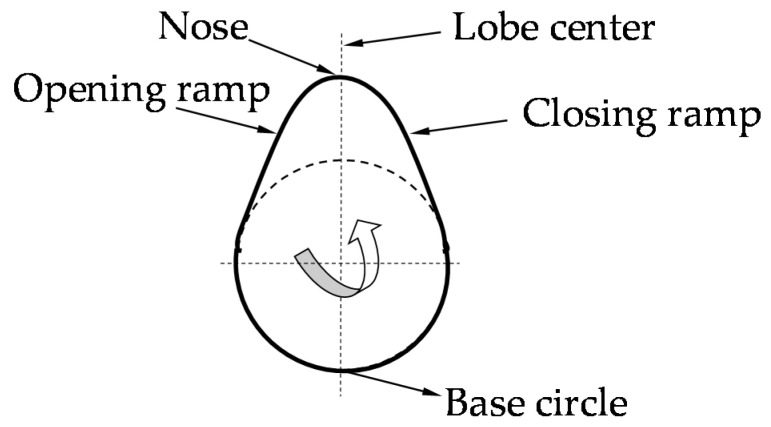
Functional surfaces on the cam.

The opening surfaces of the cams (Figure 5, opening and closing ramps) appear to be problem free, because the largest forces act on the surfaces of the cams due to the acceleration of the valves [17]. Due to the large radius of the curvature of the cam, the surfaces of the cams and the tappet are sufficiently separated by the lubricating film, which is also due to the high entrainment speed of the lubricant [18,19,20].

To verify our results in the microhardness analysis, we also performed experiments on a new camshaft, which is supplied as a spare part for the monitored engine.

**Figure 6:**
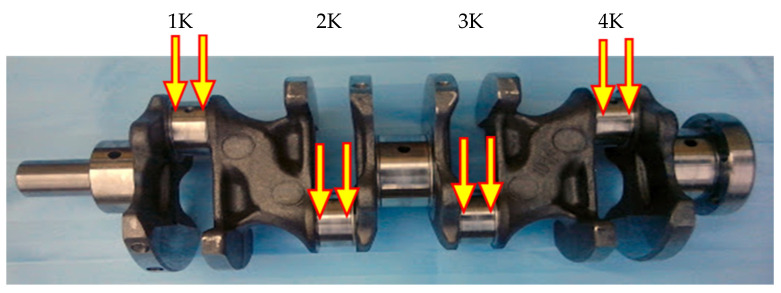
Sampling spots of connecting rod pins (1 cylinder—1K, 2 cylinder—2K, 3 cylinder—3K, 4 cylinder—4K).

In the case of the connecting rod pins, we chose to take samples from the places shown in Figure 6, since it is a rotating part of the crankshaft stressed constantly around the entire circumference. There is no rupture of the lubricating film due to the geometry of the individual components, which results in uniform wear of the entire friction surface. The selected sampling point on the connecting rod pin was also chosen due to the fact that the most common place of crankshaft failure is wear in the place of the connecting rod pin [11,21,22,23].

The samples we used in the experiments were obtained by wet abrasive cutting. A diamond cutting disc was used to separate the material in the presence of a coolant, one of the properties of which is to prevent thermal influence on the structure of the samples and, at the same time, to wash away the sawdust from the cutting surface.

In the experiments, we performed 5 measurements and evaluated the arithmetic mean. The variance of these values was up to 5%. For the objectivity of the measurement (thermal influence at the cutting point), it was not possible to carry out more provable measurements at the given place.

Sample preparation was performed using the standard method for metallographic analysis by pouring the samples into acrylic Struers-CitoFix Powder. This fixing com-pound is easy to apply with a short curing time and slight shrinkage. The cured material is thermoplastic and chemically resistant with average abrasion resistance [12].

**Figure 7:**
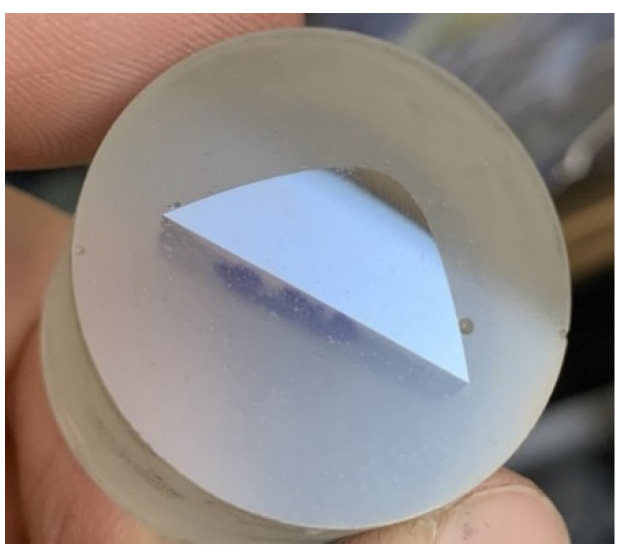
Sample ready for microhardness measurement.

The curing time was about 10–15 min. Subsequently, the samples were ground on a Struers Labopol 5 metallographic polisher (Struers, Ballerup, Denmark). By grinding the samples, the material was gradually removed from the surface of the samples using SiC grinding wheels with Al_2_O_3_, B_4_C, ZrO_2_, Si_3_N_4_ or diamond particles. Polishing wheels with a diamond paste with a grain size of 1 µm were used for the final treatment of the samples. Finally, the samples were treated by chemical etching, using a Nital etchant at a concentration of 1% with an exposure time from 15 to 30 s. Microhardness was determined on the samples thus prepared (Figure 7).

## 3. Results

### 3.1. Camshaft Roughness Measurement Results

**Figure 8:**
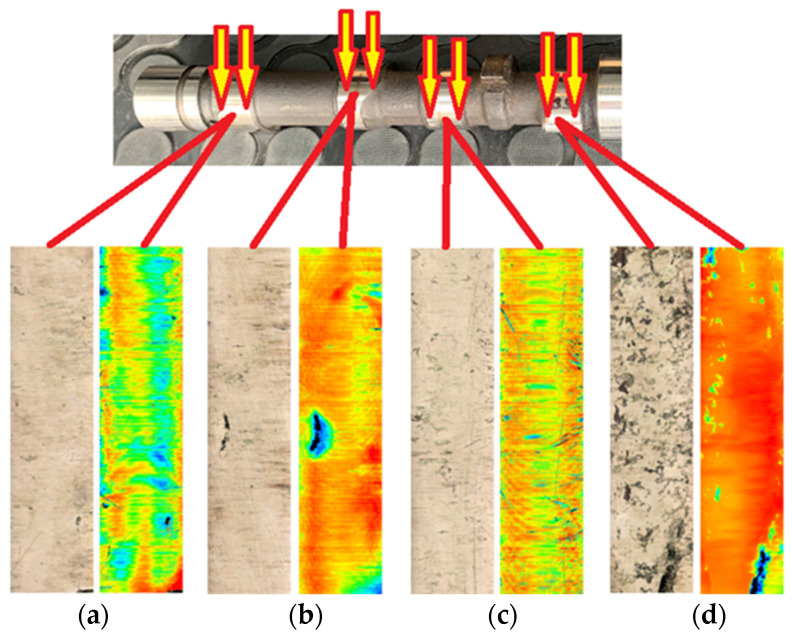
Wear of cam friction surfaces on 1st and 2nd cylinders: (**a**) 1st cylinder intake cam; (**b**) 1st cylinder exhaust cam; (**c**) 2nd cylinder intake cam; (**d**) 2nd cylinder exhaust cam.

**Figure 9:**
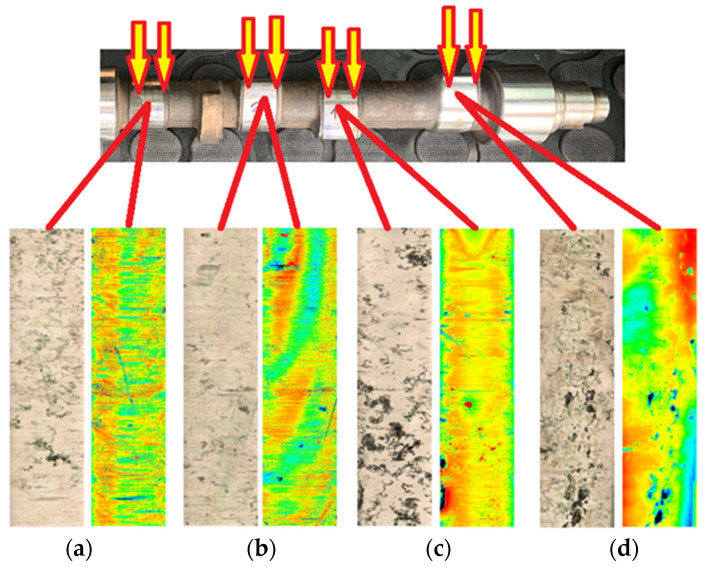
Wear of cam friction surfaces on 3rd and 4th cylinders: (**a**) 3rd cylinder intake cam; (**b**) 3rd cylinder exhaust cam; (**c**) 4th cylinder intake cam; (**d**) 4th cylinder exhaust cam.

The wear of the friction surfaces of the monitored cams is shown in Figure 8 and Figure 9. A confocal microscope with laser 3D scanning was used for the experiments.

In the analysis of the observed wear, which arose around the cam nose, its occurrence was due to rupture of the lubricating film due to insufficient lubricant feed rates, but it was also due to the small radius of the curvature of the cam nose.

The colored parts in Figure 8 and Figure 9 present the unevenness caused by wear in the friction node, where the green color presents the smallest surface roughness and the dark blue color presents the largest unevenness (scratches). The black sections on the analyzed cam surface representations show the fatigue wear that causes the pitting of the material from the cam surface. These broken particles of the material surface bond with the lubricant, further causing abrasive wear, which can be observed on the friction surfaces as scratches. These particles in the lubricant all around bomb the already damaged friction surfaces of the cams and help to further break the material, causing excessive wear.

**Figure 10:**
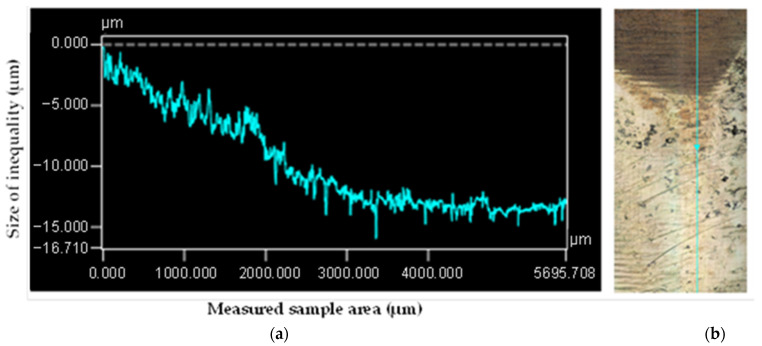
Roughness profile of the exhaust cam surface on the second cylinder: (**a**) measured values and (**b**) laser trajectory at the measuring point.

From the roughness measurement experiments and the color spectrum displayed in Figure 8, it can be stated that the greatest roughness of the surface, i.e., the greatest wear, was on the exhaust cam of the second cylinder (Figure 8d). Our finding is also supported by the course of the roughness profile on the exhaust cam of the second cylinder, as shown in Figure 10a,b, where the maximum differences of the micro-irregularities of the monitored cam reached almost 15 μm. When analyzing the other cylinders, the courses of the roughness profiles were not so significant.

### 3.2. Results of Measurements of Roughness of Connecting Rod Pins

**Figure 11:**
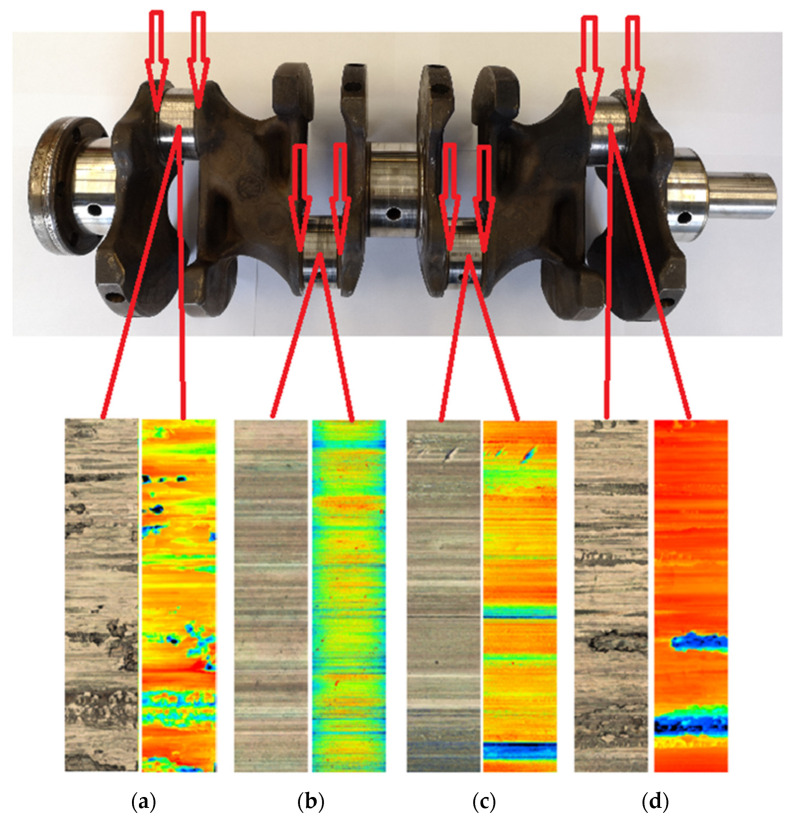
Analyzed areas of crankshaft roughness: (**a**) 1st cylinder; (**b**) 2nd cylinder; (**c**) 3rd cylinder; (**d**) 4th cylinder.

Figure 11 presents an analysis of the samples of the crankshaft connecting rod pins, from which we can state that the sample from the fourth cylinder (Figure 11d) shows the greatest wear. Our statement also supports the color spectrum of the analyzed roughness surfaces of the friction nodes, where a confocal microscope with laser 3D scanning was used. The analyzed areas, which contain a minimum of green areas, also present minimal wear. 

The color spectrum in Figure 11a,d presents visible fatigue wear, the so-called pitting—broken parts of the material from the surfaces on the first and fourth cylinders, which we could also observe on the camshaft.

The samples in Figure 11b,c, according to the color spectrum, are clearly less worn. Based on the color spectrum, the abrasive wear of the surfaces of the connecting rod pins could be caused by the tearing of a particle of material in the lubricating medium, which subsequently acted as an abrasive.

### 3.3. Results of Microhardness Measurements

#### 3.3.1. Camshaft

The functional surfaces of the camshaft are hardened, which is characteristic of this type of component made of C55 steel. The hardening temperatures are 790–830 °C for water hardening and 800–840 °C for oil hardening, and the tempering temperatures are 530–670 °C. We used the above facts to analyze the microhardness of the monitored camshaft [17]. 

**Figure 12:**
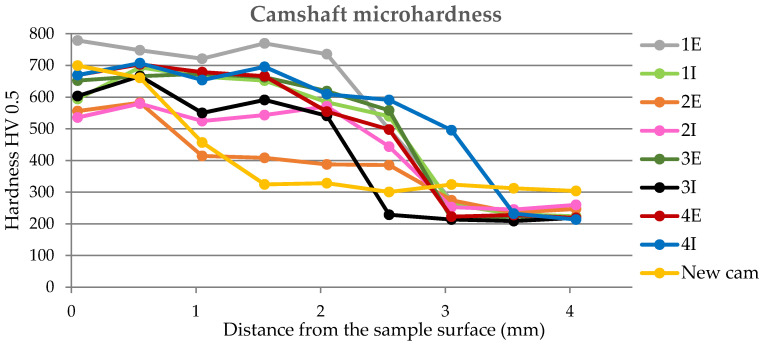
Camshaft microhardness curves (HV 0.5).

The microhardness curves indicate the fact that the thickness of the cured layer of the functional surfaces of the cams is approximately 2.5 mm, which is also evident from the graphic representation in Figure 12. From the measured values of microhardness, it follows that there is a martensitic structure on the surface of the components, which is characteristic for surface hardening, where the goal is to achieve a high surface hardness and a tough core. The hardness range in our experiments is about 540–800 HV.

**Figure 13:**
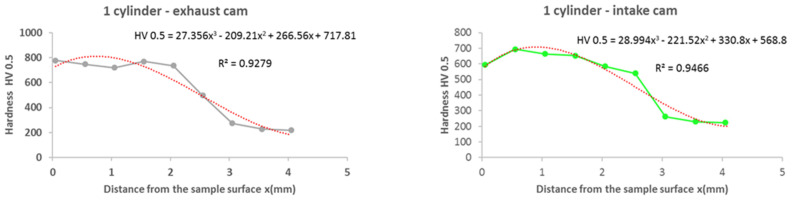
Regression analysis for a 3rd−order polynomial for the values of the 1st cylinder on the exhaust and intake cams.

**Figure 14:**
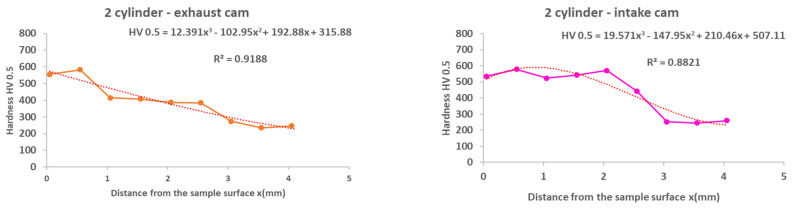
Regression analysis for a 3rd−order polynomial for the values of the 2nd cylinder on the exhaust and intake cams.

**Figure 15:**
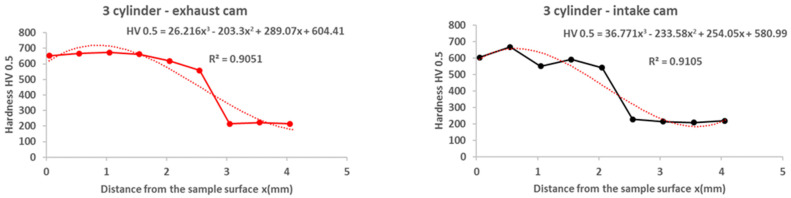
Regression analysis for a 3rd−order polynomial for the values of the 3rd cylinder on the exhaust and intake cams.

**Figure 16:**
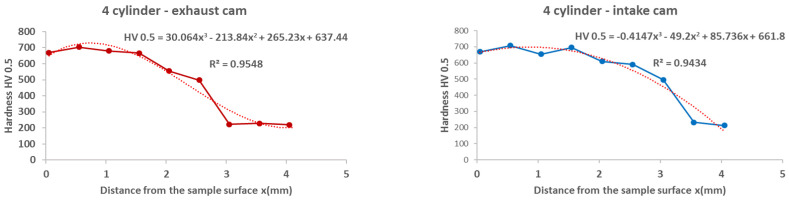
Regression analysis for a 3rd−order polynomial for the values of the 4th cylinder on the exhaust and intake cams.

**Figure 17:**
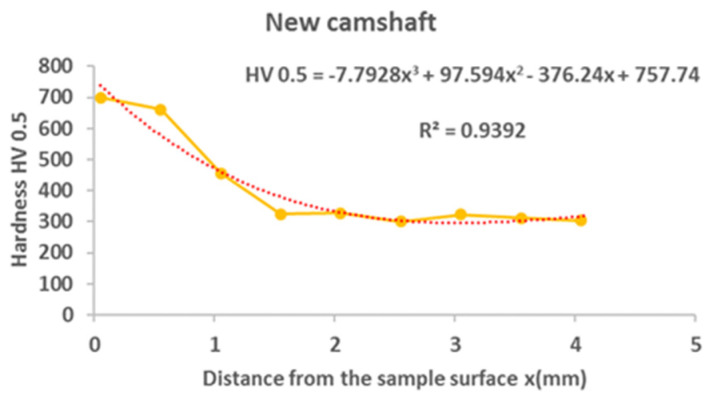
Regression analysis for a 3rd−order polynomial for the values of a new camshaft.

From the results of the microhardness of the camshaft, which was used in the experiments, as well as the new camshaft, which is supplied as a spare part from the type production for the given type of engine, we can state the following facts:

- In the course of the microhardness of the cams, in comparison with the values of the new camshaft, the hardened layer on the worn cams is significantly thicker, up to 1.5 mm, which is also evident from the graphical representation shown in Figure 12.
- The smallest measured hardness (556 HV0.5) was found for the exhaust cam from the second cylinder. Given these facts, we can state an undesirable connection of the pitting with the lubricant, where the lubrication failed similarly, as with the crankshaft. Subsequently, due to insufficient lubrication, the surface of the material overheated to the tempering temperature, and, thus, the hardness of the cams decreased.
- From the graph in Figure 12, it is possible to see the zone of the heat-treated layer with the transition to the hardness of the base material of the individual cams. Deviations in the position of the residual microhardness are affected by the degree of wear of the individual cams.
- It can also be stated that the technology of production of the old and the new cams was not identical, as the new cam has a significantly thinner hardened layer, which ultimately leads to faster wear in the engine friction nodes. These facts are also apparent from Figure 12.
- Using regression analysis, we experimentally demonstrated from a mathematical point of view that the hardness can also be expressed by polynomial function 3 of a row with a high value of the coefficient of determination R^2^, which is shown in Figure 13, Figure 14, Figure 15, Figure 16 and Figure 17, where the regression equations for the individual cylinders are also given, for the exhaust and intake cams. These statements show that the polynomial regressions used indicate a decrease in the microhardness of the monitored objects, and a more pronounced forest is observed on the new camshaft.

From the following waveforms of the individual cams of the monitored camshaft (Figure 13, Figure 14, Figure 15, Figure 16 and Figure 17), it is clear that the hardness of the heat-treated layer of the functional surfaces is about 2.5 mm, and after this value, the wear increases. In the case of a new camshaft from secondary production, the area of hardness loss is at the limit of 1.0 mm, which represents a thinner heat-treated layer, and the same surface hardness is not achieved as in the case of a camshaft from primary production. We also express this statement by a mathematical expression using 3-rd order polynomial regression dependences with a high value of the coefficient of determination R^2^. 

#### 3.3.2. Crankshaft

The functional surfaces of the crankshaft are tempered by hardening, which is characteristic of this type of product made of steel 37Cr4. The hardening temperatures are 800–860 °C for water hardening and 840–880 °C for oil hardening, and the tempering temperatures are 160–180 °C. We used the above facts to analyze the microhardness of the monitored crankshaft [17].

**Figure 18:**
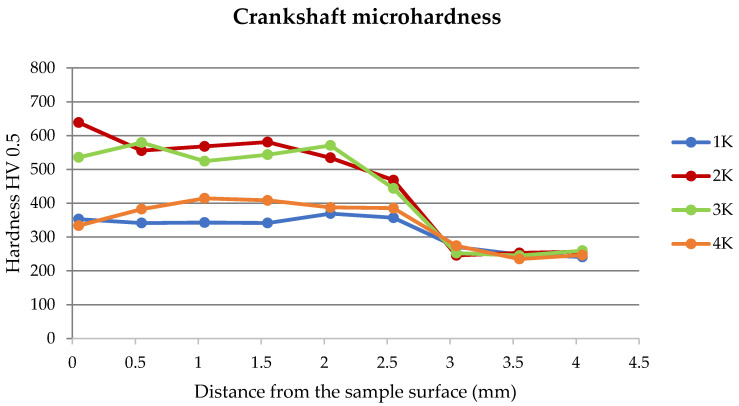
Crankshaft microhardness curves.

From the measured microhardness of the monitored samples of crankshaft connecting rod pins (Figure 18), the surface hardening due to the improved material properties on the surface of the monitored components is about 2.5 mm thick, which is evident from Figure 18. However, some authors state that, in the final surface treatment after grinding and in the renovation of crankshafts made of other materials, a thickness of 2 mm is sufficient, e.g., using the ultrasonic nanocrystal surface modification (UNSM) method. At this thickness, the original fatigue strength is restored, and the tribological properties of the friction node are restored [12].

Our experiments showed microhardness values on the surface in the range of about 530–640 HV. We can therefore state that the values proving the hardened layer were found only on the second and third cylinders. Cylinders 1 and 4 had significantly lower microhardness values of only 350–400 HV, which indicates that lubrication failure and subsequent overheating of the material above the tempering temperature could have occurred in these parts. Subsequent gradual cooling of the material led to the tempering of the material mainly in those places where lower hardnesses of the material were measured by the experiments, which is evident from Figure 18.

**Figure 19:**
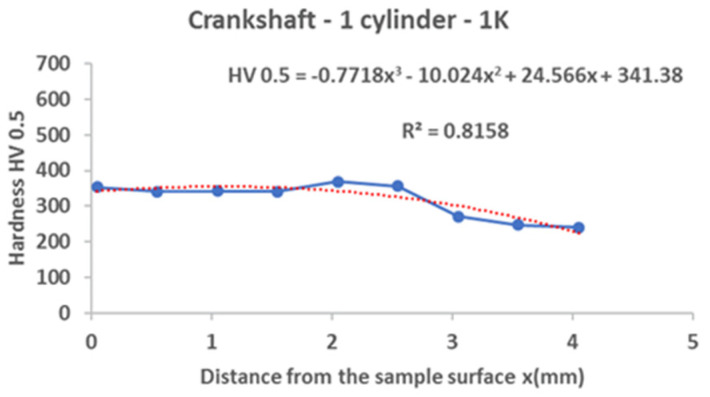
Regression analysis for a 3rd−order polynomial for crankshaft values on 1st cylinder.

**Figure 20:**
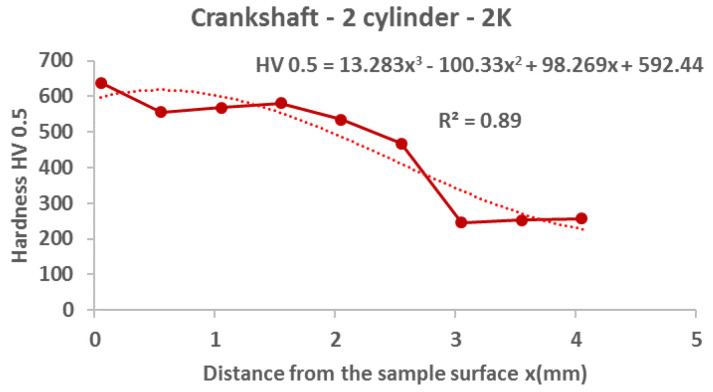
Regression analysis for a 3rd−order polynomial for crankshaft values on 2nd cylinder.

**Figure 21:**
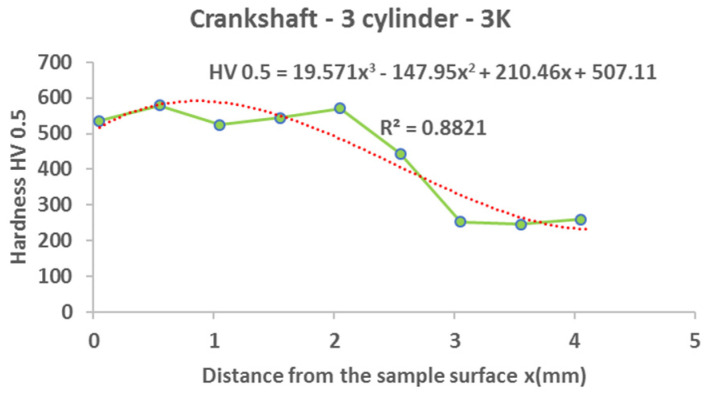
Regression analysis for a 3rd−order polynomial for crankshaft values on 3rd cylinder.

**Figure 22:**
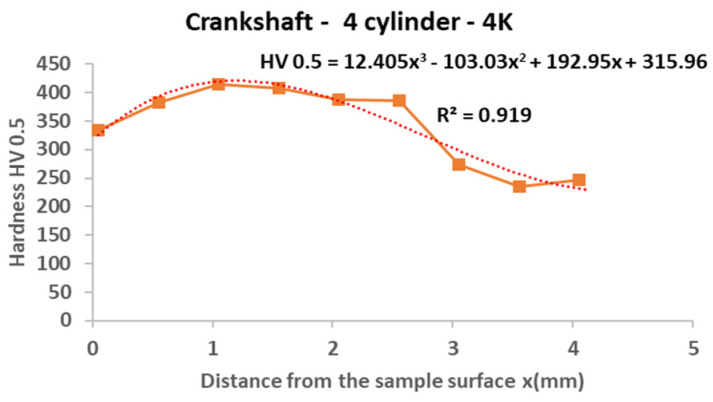
Regression analysis for a 3rd−order polynomial for crankshaft values on 4th cylinder.

From the following graphs of the monitored crankshaft (Figure 19, Figure 20, Figure 21 and Figure 22), a zone of the heat-treated layer (approx. 2.5 mm) with a transition to the hardness of the base material is evident. Deviations in the position of the residual microhardness are influenced by the degree of wear of the individual functional surfaces of the shaft. We also express this statement by mathematical expression using third-order polynomial regression dependences with a higher value of the coefficient of determination R^2^.

### 3.4. Results of Microscopic Analysis

When verifying the experiments from the point of view of the surface microstructure, with a new cam and the used cam, we used a confocal microscope with laser 3D scanning, which is suitable not only for monitoring the roughness profile but also for observing the surface microstructures. With this experiment, we wanted to confirm our hypothesis that the technology of production of the old and new cams is not identical, as the new cam has a significantly thinner hardened layer, which ultimately leads to faster wear in the engine friction nodes.

**Figure 23:**
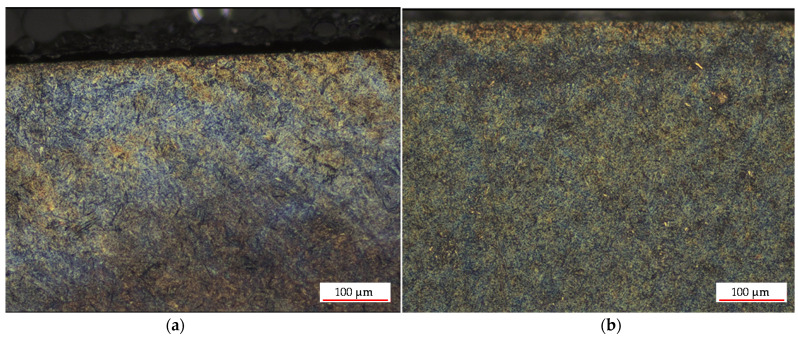
Surface microstructure: (**a**) new cam and (**b**) used exhaust cam 2nd cylinder with the smallest microhardness.

The surface microstructure in Figure 23 is a visible martensitic structure, which verifies surface hardening as a heat treatment to obtain a harder cam surface. Comparing the structures in Figure 23 of the new cam and the used cam with the lowest hardness at the second cylinder, it can be observed that the martensitic structure of the new cam has thicker martensite needles. The surface microstructure of the new cam consisted of quenched martensite and that of the used cam of tempered martensite. 

**Figure 24:**
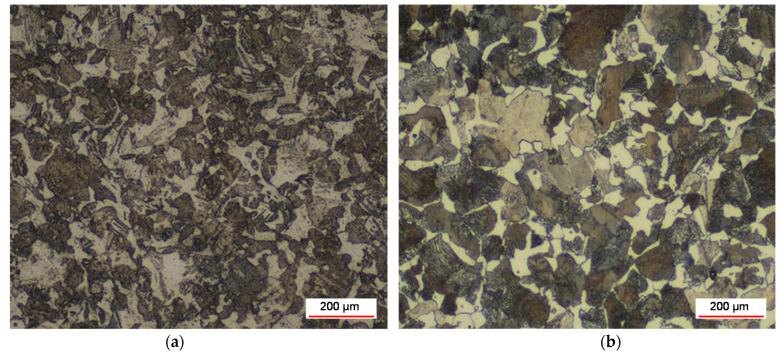
Camshaft core: (**a**) new cam and (**b**) used exhaust cam 2nd cylinder with the smallest microhardness.

From the monitoring of the microstructure of the core of the basic material of the camshaft C55, shown in Figure 24, we can state that it is a pearlitic–ferritic structure, the purpose of which is to maintain the flexibility of the camshaft. In the area up to 2.5 mm from the hardened cam face, the original austenitic grain is bounded by a ferritic mesh. As the distance from the forehead increases, the amount of ferrite increases. Increasing the proportion of ferrite and coarsening the perlite cause a decrease in hardness, which appears to be an undesirable phenomenon when using a camshaft.

## 4. Conclusions

In this paper, we analyzed the functional surfaces of the rotating parts of the friction nodes in an internal combustion engine. The results of the experiments show that the surfaces of the rotating parts showed visible wear on the cams and connecting rods. We conducted experiments to look for possible causes of the wear.

The following findings were obtained:

- The monitored components of the camshaft, on which we measured microhardness values of less than 550 HV on the surface, were clearly heated above the tempering temperatures of the materials. Based on the above facts, due to the heating of the material to a high temperature, there was a subsequent gradual cooling and tempering of the material. The tempering caused the material structure to soften to lower microhardness values, which is unacceptable mainly due to the service life of the monitored friction nodes. A lower value of microhardness in the zone of the heat-treated layer, with the transition to the base material, is one of the possible causes of wear, which we observed on the surfaces of the friction nodes. The wear of rotating parts results in complex mechanisms and various other factors that must be taken into account.
- From the measured values of microhardness according to Vickers, it can be observed that the exhaust cam from the second cylinder was so thermally affected that the material was tempered by the effect of which the hardness decreased to a value of up to 556 HV. Compared to the new cam, whose hardness reached a value of about 700 HV, the hardness drop in the new cam was rapid and incompatible with the prescribed values of the hardened layer, where the heat-treated layer was up to 1.5 mm thinner than that of the original camshaft from primary production.
- Material loosening also occurred on the connecting rod pins of the crankshafts at cylinders 1 and 4. Here, the values of microhardness decreased due to the tempering of the material to values of about 350 HV. Compared to cylinders 2 and 3, which achieved microhardness values in the range of 550–640 HV, this is an extraordinary decrease of almost 50%. The roughness analysis of the connecting rod pins confirmed the greatest wear, which was found on the cylinders with the lowest hardness, i.e., the first and fourth cylinders.
- When comparing the microhardness of the worn cams on the used camshaft and the new camshaft, it can be observed that the hardened layer of the new cam from the secondary production has a significantly thinner thickness compared to worn cams. From this finding, it can be stated that spare parts from secondary production are not produced in the same quality or with the same technology as the original parts from primary production.
- We also found support for the difference between the production technology in the production of the used and the new camshafts by carrying out a microscopic analysis of the materials of the examined objects. The new cam has a significantly thinner hardened layer, which ultimately leads to faster wear in the engine’s friction nodes.
- We also supported our findings with a mathematical expression of the measurements of individual cams and connecting rod pins. From the mathematical aspect, we experimentally proved the suitability of using regression analysis; thus, the hardness can be expressed by a polynomial function of the third row, where the results have a high value of the coefficient of determination R^2^. These statements show that the polynomial regressions used indicate a decrease in the microhardness of the monitored objects, and a more significant decrease was observed on the new camshaft.
- Based on all the results from the experiments, it is possible to expect more comprehensive information, especially in terms of roughness and microhardness of the materials used for functional pairs of friction nodes, which can help in the study of suitable materials for the production of monitored components used in automotive industry.
- One aspect of our conclusion points to the fact that, in the production process, it is necessary to follow the production procedure, which, in this case, is the chemical/thermal treatment of functional parts in order to avoid different properties of surface layers. The production of spare parts (either of base materials or of surface-reinforced layers) from secondary production should have the same qualitative features. Another aspect of our conclusion is the recommendation to shorten the oil change intervals to a maximum of 10,000–15,000 km in heavy traffic (urban cycle) or to apply oil additives enabling the solubility of combustion contaminants already in new vehicles, thus reducing the possible contamination of the oil by abrasive flue gas particles. These main aspects would significantly reduce the wear of functional pairs and prolong the technical lifespan of engines.

## Figures and Tables

**Table 1:** Parameters of the engine Skoda 1.4 MPi.

Parameters	Values
Stroke volume	1397 cm^3^
Power	50 kW at 5000 min^−1^
Torque	120 Nm at 2500 min^−1^
Fuel type	Natural 95 (E10)
Mixture preparation	Multi Point Injection
Number of cylinders	4
Number of valves	8
Engine timing	OHV
Engine type	I
Engine mounting	front across

**Table 2:** Chemical composition of the material C55 (%).

C	Mn	Si	P	S	Cr	Ni	Cu
0.52–0.6	0.5–0.8	0.15–0.4	≤0.04	≤0.04	≤0.25	≤0.3	≤0.3

**Table 3:** Mechanical properties of the material C55.

Yield Strength (MPa)	Ultimate Strength (MPa)
480	750–900

**Table 4:** Chemical composition of the material 37Cr4 (%).

C	Mn	Si	P	S	Cr
0.35–0.42	0.5–0.8	0.17–0.37	≤0.04	≤0.04	0.8–1.1

**Table 5:** Mechanical properties of the material 37Cr4.

Yield Strength (MPa)	Ultimate Strength (MPa)
785	981–1177

## Data Availability

The data presented in this study are available on request from the corresponding author.
